# Pediatric dentists’ perspectives of children with special health care needs in Japan: developmental disabilities, phobia, maltreatment, and multidisciplinary collaboration

**DOI:** 10.1186/s12887-021-02711-2

**Published:** 2021-05-19

**Authors:** Ayako Ide-Okochi, Hiromi Funayama, Yoshinobu Asada

**Affiliations:** 1 Department of Nursing, Graduate School of Health Sciences, Kumamoto University, 1-24-4, Kuhonji, Chuo-ku, Kumamoto City, Kumamoto Prefecture Japan; 2 Dep. of Pediatric Dentistry, School of Dental Medicine, Tsurumi University, 3-1-2, Tsurumi, Tsurumi-ku, Yokohama City, Kanagawa Prefecture Japan

**Keywords:** ASD, Neglect, Dental phobia, Health literacy, Health checkup, Mother, Dilemma, Multidisciplinary collaboration

## Abstract

**Background:**

The number of children diagnosed with developmental disabilities (DDs) or other chronic difficulties has risen. However, each professional’s awareness of children with developmental, emotional and behavioural difficulties may differ, allowing their special needs to be overlooked at child health checkups until secondary difficulties appear. Therefore, it is necessary to explore the multi-professional views of children with such chronic difficulties. This study investigates pediatric dentists’ perception of children with potential chronic difficulties.

**Methods:**

Interviews were conducted with 21 pediatric dentists, and the transcripts were analyzed using grounded theory to develop categories for the theoretical assessment.

**Results:**

Four themes emerged regarding the children with potential chronic difficulties: children exhibiting possible DDs with awkward social communication and interaction; severe rampant caries possibly derived from maltreatment; dental phobia possibly derived from mental health problems; a complicated home environment where their mothers exhibit poor oral health literacy.

**Conclusions:**

This study’s findings imply that participants’ concept of children of concern included the risks of poor oral health and mental health problems that other healthcare professionals might overlook. It is recommended that multidisciplinary professionals engaging in child health checkups be aware of children’s oral and mental health status as well as potential DDs and child maltreatment.

## Background

Improving maternal and child health has been a global theme of community health care systems [1]. In Japan, the national campaign promoting maternal and child health launched in 2001 [2]. Since the secondary phase of this campaign, it decided on two prioritized agendas: 1) support tailored to parents who have difficulties raising their children; 2) prevention of child abuse from pregnancy [2]. Both difficulties with raising children and child abuse are likely to be derived from the mixture of child, parent, and sociocultural, environmental factors. Among various factors, children with developmental disabilities (DDs) and their parents’ stress were considered [3, 4]. A systematic review and meta-analysis revealed that parenting stress levels were higher for parents of children with ASD/DD than parents of children from other clinical groups [5]. A meta-analysis also found that parenting stress was a significant risk factor for child abuse and neglect [6]. Therefore, it is essential to detect children with the possibilities of developmental difficulties or child maltreatment in the early stage and provide care to those children and their parents.

However, the rate of children with supposed developmental difficulties was considerably different among the municipalities. An annual report indicated the rate of children with abnormalities differed with a range of less than 5% to more than 60% in the Tokyo area [7]. Therefore, it is essential to promote equalizing skills of multidisciplinary professionals who engage in legitimate child health checkups to prevent overlooking children’s developmental risks [3].

The term *kininaru-kodomo* (children of concern) has been widely utilized in Japan to refer to children regarding whom professionals are concerned about the possibility of mild developmental disorders (DDs); child maltreatment; and other behavioural, emotional, or social problems without any diagnosis or official record of individual or environmental problems [8]. This term is practical because multidisciplinary professionals share their concerns about children and parents even before their concerns were determined as a diagnosis or legal child protection [9].

However, previous studies have mainly explored the perception of children of concern among public health nurses and daycare centre teachers [8–10]. According to the type of licenses and the following experiences, inter-professionals tended to have specific viewpoints on children’s risk factors and rights so that the philosophies of care coordination are sometimes not unanimous [11, 12]. Among three legitimate healthcare professionals engaging in the legitimate child health checkups in Japan, it was reported that public health nurses tended to mind the signs of child abuse and neglect, while doctors mainly watched out the health overall [3]. Nevertheless, it has not been explored how pediatric dentists perceive children’s possible health risks, although they are one of the three legitimate healthcare professionals at legitimate child health checkups.

According to a national survey which collected school teachers’ report, 6.5% of standard class students in Japan are considered as having mild DDs, that is, autism spectrum disorder (ASD), attention deficit hyperactivity disorder (ADHD), and learning disorder (LD) [13]. According to the number of children with possible DDs, 13.3% of 3-year-old preschoolers were considered to have difficulties derived from DDs, according to preschool teachers’ report [10]. Moreover, in Japan, 122,575 cases of child abuse were reported in 2016, increasing from 56,384 in 2010 [14]. Beyond Japan, globally, children with DDs consists of a significant minority [15]. In the United States, the Centers for Disease Control and Prevention admitted that approximately one in six children aged 3–17 years had one or more developmental or behavioural disabilities [16]. In the United States alone, nearly 1,000,000 children are victims of nonaccidental trauma annually [17]. Therefore, it is urgent to adequately screen children with possible health risks by clarifying each professional’s perspectives engaging in child health care.

However, dentists’ perception of children of concern has been little studied.

Oral health probably reflects the existence of children’ s DDs and the parents’ parenting attitudes. Children with special health care needs (CSHCN) experience more considerable challenges with oral care in the home and dental office than their typically developing peers [18, 19]. A previous study in India showed that oral healthcare needs remained unmet, although the prevalence of dental caries was high among children with special health care needs [18]. Moreover, mothers at dental clinics do not dare hide their children’s disabilities and their maltreatment [19, 20]. Therefore, poor oral health is likely to indicate children’s potential disabilities and parents’ neglectful attitudes toward their children. That is why dentists could contribute to identify children’s potential chronic problems derived from individual and environmental factors. Furthermore, considering the professional’s responsibility for child abuse prevention [21], there is an urgent need to explore what aspects pediatric dentists felt alerted. Therefore, this study aims to elucidate the pediatric dentists’ perceptions of children of concern.

## Methods

Qualitative research is suitable to explore the phenomenon that involves interactions among people in the research field [22]. Among qualitative study designs, the grounded theory approach was selected over other designs because it enables concept development through constant data comparison. According to the grounded theory approach, the theory is distinguished from the description and is also an arrangement of systematically interrelated concepts, thereby explaining the phenomenon. This research also intended to clarify the relationships among concepts. The data were collected through individual interviews that were audiotaped, and transcribed. Observations were recorded in field notes.

After receiving the Human Research Ethics Committee’s approval, the second author HF recruited potential participants at a pediatric dentistry outpatient clinic in a university hospital. This university hospital is located in A district of B city with a population of 3.7 million, which is the largest among cities in Japan. A district is closest to Tokyo and where the number of Okinawan and Brazilian residents is relatively high. Moreover, this university hospital’s management body runs a mother and child life support shelter next to the hospital. Because of proximity and support needs, pediatric dentists who belonged to the studied clinic had offered yearly dental health checkups at the shelter mentioned above. Therefore, participants were likely to have considerable knowledge and experiences related to child abuse. Furthermore, it was hypothesized that participants had positive attitudes toward child abuse prevention because early detection of abused children was their agenda [23].

The studied clinic had approximately 30, relatively many members compared to other pediatric dentistry departments in Japan. Also, two-thirds of members were females. Female dentists constitute approximately 40% of the Japanese Society of Pediatric Dentistry members [24]. Compared to the rate mentioned above, this clinic had more female members because of the supportive policy toward female dentists, who had difficulties in continuing their work but were eager to contribute to maternal and child health [24].

Twenty-one dentists with 2–32 years of occupational experience were included to collect various viewpoints. Sociocultural factors affect the attitudes toward child abuse, and for example, Iranian pediatric dentists had tolerant views on violent discipline customs [25]. According to previous studies, the clinician’s educational level on the job training and treatment experience is not unanimous regarding children with developmental disabilities, child abuse, and neglect [19, 26]. Therefore, it was considered essential for including various pediatric dentists in terms of age and credentials to explore a genuine concept of children of concern. Table 1 shows the demographic characteristics of the participants. Although younger participants did not have pediatric dentistry credentials, we refer to participants as pediatric dentists in this paper. There included 3 Board Certified Trainers and 3 Board Certified Fellow of the Japanese Society of Pediatric Dentistry. In addition to working at the studied clinic, participants had also worked as part-time workers at private dental clinics and public healthcare centres.

**Table 1:** Demographic characteristics of the participants (*N* = 21)

Characteristics	Number
Gender	
Female	14
Male	7
Age	
20s	6
30s	10
40s	2
50s	3
Years of current job experience	
1 year–4 years	7
5 year–9 years	5
10 year–14 years	4
15 year–19 years	2
30 year- and above	3
Board Certified Trainer of the Japanese Society of Pediatric Dentistry	3
Board Certified Fellowof the Japanese Society of Pediatric Dentistry	3

Potential participants received a query letter which requested a response from those interested in being interviewed. The query letter included a brief description of the study and asked for participation based on their willful decision. Purposeful sampling is one of the major principles of this methodology [27] to maximize the opportunity to develop new concepts and interrelate existing concepts. The first participant, who had both pediatric and disability dentistry credentials, told multiple aspects of children of concern closely related to the possibilities of developmental disabilities. The codes reflecting the first participants’ notion of children with disabilities were developed, and then the variations among the codes related to disabilities were sought. The next interviewee was recruited based on developed concepts, while constant comparisons among concepts were made during the intertwined phases of data collection and analysis [22]. After a discussion among authors, the second author HF and third author YA recommended the next interviewee based on a list of potential participants. Through the interviews of 21 participants, data were considered saturated when no more codes could be identified, and there were enough variations to explain categories. All the participants were offered a gift card of 1000 Japanese yen (less than 10 $).

A semi-structured interview was used to explore the perception of children of concern from an insider’s perspective. The interview guide was constructed through a literature review regarding children of concern [8] and the opinion of second author HF and third author YA who had credentials of pediatric dentistry and the third author YA served as the president of Japanese Pediatric Dentistry. The interview questions included the following: (a) what is the definition of children of concern? (b) how are mothers of children of concern? (c) what are the difficulties in dealing with children of concern? (d) what are the strategies of health promotion of children of concern? Although these questions were guided in a semi-structured interview protocol, the questions were increasingly focused and detailed in subsequent interviews. Interviews were conducted in a private room located on another floor from the studied clinic from May 2015 to August 2016. The principal researcher AO also conducted fieldwork from January 2015 to October 2018 at the studied clinic and the maternal and child living support facility, observing the participants’ interactions with the patients and mothers.

The analysis employed in the grounded theory approach involves open, axial, and selective coding to develop analytic categories. Open coding divides data into meaningful parts (codes) to make categories so that similar codes are gathered and compared continuously to develop more abstract concepts. Axial coding is utilized to interrelate categories to maximize the potential of developing theories. Selective coding is used to develop core categories that integrate whole categories and provide cohesive explanations of phenomena. Using computer software for qualitative data analysis enables more transparent, rigorous qualitative analysis [28]. NVivo 11 was employed during the process of developing categories. Moreover, the data were considered saturated when no more codes could be identified, existing categories were coherent, and enough variations to explain them. The second author HF and the third author YA critically reviewed the analysis process and interpretation.

The present study followed the method that Guba and Lincoln [29] recommended enhancing validity. One participant with over 30-year occupational experience checked a summary of emergent themes and assured that the interpretation was accurate to their perceptions. An expert in qualitative research also checked that the data and interpretations were coherent and audited the study process. Additionally, the principal researcher AO had known many participants and has cooperated in providing health education to children and mothers who reside in a shelter. Moreover, maximizing the length of job experience assured that the data variations are the rationale for triangulation.

Research approval was obtained from the Tsurumi University Research Ethics Committee (Approval no: 1303, 9 May 2015; 1511, 20 July 2017). All the participants were informed of the study’s objective and design, and written consent was obtained from the participants for interviews. They were also free to leave the interview if they wished.

## Results

**Fig. 1:**
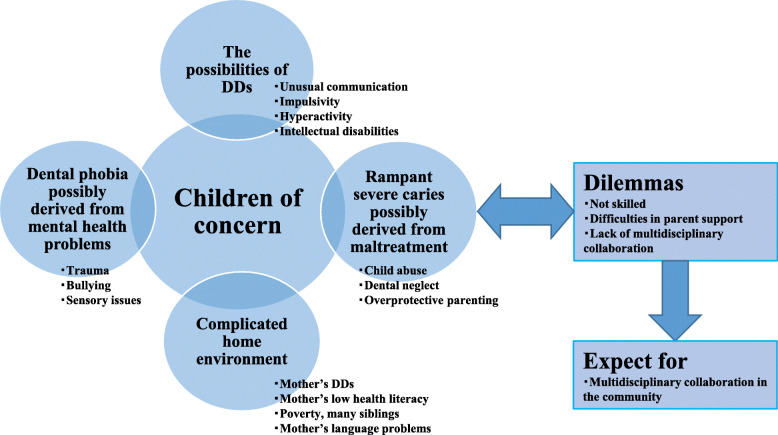
Core categories of participants perception of children of concern

The average length of interviews was 29 min. From the qualitative analysis with NVivo, the average number of nodes and references per participant was either 18 and 30. In this paper, we use the word nodes and codes and references and quotes interchangeably. The data analysis revealed a conceptualization of how pediatric dentists perceive children of concern, paying attention to children’s health status and well-being, with four core categories, DDs, dental phobia, rampant caries derived from child maltreatment, and complicated family environment (see Fig. 1). Regarding DDs, possible ASD, mentally retarded, and impulsive and hyperactive behaviours were the topics participants said they cared about (see Table 2). Due to behavioural difficulties during treatment, participants felt that the treatment of children of concern was complex.

**Table 2:** Children of concern as perceived by the participants

Core categories	Categories	Examples of quotes (participant ID No.)	Participants' ID No. with related quotes
Children who exhibit the possibilities of developmental disabilities (DDs)	Children who exhibit unusual communication	We do not communicate well with each other, but children keep talking by themselves. I wonder if they are talking because they are afraid of something or if they are also constantly talking at home (No. 13).	No. 1, 2, 3, 8, 10, 12, 13, 16, 20, 21
	Children who exhibit impulsivity	A child who does not listen to me at all and cries and acts violently. One kid got up suddenly when I was making a mould and went into the trash (No. 5).	No. 1, 3, 4, 5, 6, 7, 9, 11, 16, 18, 19
	Children who exhibit hyperactivity	When I call the patient to come in, other children come to me, greet me as customary, and can reply with, "Yes." However, children with concerns never come in such a natural way. As soon as I notice such children have come, they immediately disappear (No. 3).	No. 2, 3, 6
	Children who have intellectual disabilities	Including children with mild MR (mentally retarded), children with autism. Generally, they have autism, and they have a wide range of disabilities (No. 14).	No. 2, 4, 5, 14, 17
Children with dental phobia possibly derived from mental health problems	Children who might have trauma	I suppose some children truly have a phobia. In fact, I do not know they have experienced something that remains as trauma (No. 10).	No. 1, 10
	Children who might experience bullying	When the patient went to elementary school, they had unknown mental health problems (No. 19).	No. 7, 19
	Children who might have sensory issues	Recently, I have noticed children who even show vomiting reflex. So to speak, I occasionally see children who may have dental phobia (No. 1).	No. 1
Children with severe rampant caries possibly derived from maltreatment	Children who might be a victim of child abuse	The condition of the cavity is not normal. When that happens, I wonder what I should do as the child is not cared for by their parents (No. 5).	No. 2, 5, 7, 10, 16, 17, 20
	Children who might be a victim of dental neglect	The patient’s mother had come to the aesthetic dentist…Even though that mother was very motivated about her teeth, many problems in her child’s mouth would really surprise you (No. 15).	No. 1, 2, 3, 12, 15, 16, 17, 18, 19
	Children under overprotective parenting	Parents are unusually fond of their child. Occasionally, strange situations happen that I feel parents are strange. Far from loving their child, they spoil their child, and therefore, their child cannot listen to instructions at all (No. 16).	No. 1, 2, 6, 8, 14, 16, 17
Children with a complicated home environment	Children whose mothers exhibit the possibility of DDs	I don’t know for sure, the mother herself has some problems, so-called autism because I can’t make myself understood when I talk to her (No. 1).	No. 1, 2, 3, 11
	Children whose mothers have low health literacy	I do not know whether such mothers have little interests or genuinely do not know. Some mothers say their children's teeth have melted down since birth. (No. 17).	No. 2, 11, 17
	Children in poverty and with many siblings	I consider that some smelly children are not the victim of neglect. However, mothers do not have time to take care of because they have many siblings (No. 2).	No. 2, 3, 12, 18
	Children whose mothers have language problems	I think that dealing with mothers who are not Japanese is difficult. I guess there is a difference. After all, cultural differences are complex (No. 18).	No. 18

Moreover, they had dilemmas in explaining to children’s parents the possibilities of DDs. Therefore, they expected the multidisciplinary team to discuss and care for children of concern. Participants mentioned the possibilities of dental phobia, trauma influences, and sensory sensitivity regarding mental health problems. When they suspected maltreatment, the signs of neglect, abusive behaviours, and overprotecting parenting were the categories of the participants’ concerns. The home environment, such as mothers’ low health literacy, rampant caries, and possible DDs, were concerned. Participants considered that multidisciplinary team care was necessary to keep children of concern and their family healthy as a whole.

### Dentists’ perspectives of children of concern

#### Children with possible DDs

The majority of participants described that children of concern showed autistic-like traits, such as echolalia, making less eye contact, and no response when their names were called. Most participants also affirmed that they felt cared about children when their conversation did not proceed well. A participant who had 7 years of clinical experiences perplexingly said, “We do not communicate well with each other, but children keep talking by themselves. I wonder if they are talking because they are afraid of something, or if they are also constantly talking at home (participant ID No. 13).” The possibility of a mentally retarded was also taken into consideration. About one-fourth of participants said that they assessed both probabilities of the comorbidity of ASD and the sole intellectual disability when they found communication with children awkward.

Moreover, a participant explained her puzzlement when she met a child who exhibited unusual impulsivity and hyperactivity. The majority of participants admitted that they cared for the possibilities of DDs when children showed unusually impulsive behaviours such as hiding in a trash box that looked tight for the children’s body. Behaviour problems such as shout, escape, kicking, beating, and excessive crying were reported as the participants’ concerns. Most participants stated their doubt of children’s having ASD when children exhibited unusual behaviours compared with typically developed children. Although the minority of participants told children’s supposed ADHD, most participants said they were more concerned about children’s supposed ASD. Also, the minority of participants mentioned that children of concern kept walking around without communication, and they could not hold the children if they did not use a restrainer.

When I call the patient to come in, other children come to me, greet me as customary, and can reply with, “Yes.” However, children with concerns never come in such a natural way. As soon as I notice such children have come, they immediately disappear (Participant ID No. 3).

Junior dentists in their 20s and 30s admitted that treating children with DDs or supposed DDs was difficult. They said that they panicked and almost lost their temper when such children could not obey their instructions, and therefore, the treatment would take time. In addition to the difficulties in treating children, most participants mentioned the difficulties in explaining parents. Although they sensed the possibilities of DDs in children of concern, they recognized that they did not have the authority to tell their concerns to the children’s parents. However, they contemplated that children of concern should be linked to developmental rehabilitation early. Therefore, they were keen on children who had not been diagnosed, although the children were suspected of having DDs. On the other hand, senior participants with more than 10-year-old clinical experiences said that they were not much concerned about the children with the diagnosis, as long as their parents accepted the diagnosis and adequately cared for them.

All participants admitted that they could not verify the reliability of their concern of children’s possible DDs unless parents share information with participants. Therefore, they regarded multidisciplinary collaboration as necessary. They expected a community healthcare system to exchange opinions with the centre for developmental disorders, community nurses, care workers, and daycare teachers.

Children with possible DDs often go to the centre for developmental disorders.

However, I guess that most mothers think it has nothing to do with dentists. I hope professionals can share such information more easily (Participant ID No. 16).

#### Children with dental phobia possibly derived from mental health problems

The minority of participants stated that they were concerned for children who showed potential for dental phobia. According to participants, dental phobia accompanied strong rejection of dental treatment, vomiting, and fainting. A participant with a credential of the Board Certified Trainer said, “Recently, I have noticed children who even show vomiting reflex. So to speak, I occasionally see children who may have a dental phobia (Participant ID No. 1).” The minority participants interpreted that dental phobia might be derived from the trauma that children of concern experienced unpleasant dental treatment previously.

Concerning trauma, a participant with a seven-year-old clinical experience mentioned that she cared for a patient who cried for hours, saying, “When the patient went to elementary school, they had unknown mental health problems (Participant ID No. 19).” The participant above hypothesized that the patient became withdrawn after bullying at an elementary school.

Moreover, another participant paid attention to sensory problems to explore the cause of excessive crying among children of concern.

As you know, some children are so sensitive that they dislike being touched the inside of their mouth (Participant ID No. 1).

#### Children with severe rampant caries possibly derived from maltreatment

The majority of participants were very wary of children with severe rampant caries. For example, there were not a few severe cases who lost almost all child teeth decayed from the root. Most participants considered the children’s rampant severe caries unusual, and therefore they became concerned about the children. In their perception, children’s rampant severe caries and frequent recurrence of those interrelate the possibilities of their parents’ abuse or neglect.

The condition of the cavity is not normal. When that happens, I wonder what I should do as the child is not cared for by their parents (Participant ID No. 5).

One-third of participants mentioned that they were concerned about the children with possible child abuse, for example, when a young child had a bruise on his or her eyes. A participant with the least years of clinical experience said that everyone at the studied clinic was shocked to see a mother yell at her boy and did not give his slacks and even threw them opposite him. Although most participants cared for the possible abuse or neglect, they were circumspect of calling the child consultation centre for child protection.

Nearly half of the participants mentioned the possibilities of neglect by their mothers: physical neglect, emotional neglect, and dental neglect. As for physical neglect, participants minded unsanitary conditions in that a mother did not change a diaper timely so that a child developed diaper rash. They also mentioned concern for children who exhibited an unbalanced diet, such as a 3-year-old patient who prepared his or her meal independently and, in fact, drank cola only for breakfast. In a participant’s remark, another 3–4-year-old patient said he or she ate a steak for breakfast, which was an unusual breakfast menu in the Japanese context. Therefore, a participant (Participant ID No. 8), who had a credential of the Board Certified Trainer, interpreted that this patient had not eaten anything for breakfast. Moreover, the dirtiness of faces, smell, and clothes’ conditions were the objects that participants watched out for.

Concerning the possibilities of neglect, nearly half of the participants noticed some mothers seemed little interested in their children’s achievements. For instance, some mothers were considered less responsive, showing no joy when participants praised the children for their perseverance during dental treatment. Nearly half of the participants noticed mothers’ indifference to their child potentially led to dental neglect. Participants listened to mothers with doubt because mothers asserted that their children said nothing about their cavity pain. A participant with the credential of Board Certified Fellow of the Japanese Society of Pediatric Dentistry was appalled at the mother’s excuse of refusing the follow-up appointment for treating her children’s rampant severe caries, saying that the mother argued that she was too busy to bring her child to the treatment and check children’s toothbrushing at home. Actually, the mother had enough time to eagerly come to an aesthetic dental clinic for whitening her teeth.

The patient’s mother had come to the aesthetic dentist … Even though that mother was very motivated about her teeth, many problems in her child’s mouth would really surprise you (Participant ID No. 15).

Overprotecting parenting attitudes was also the factor that participants cared for related to poor dental health among children of concern. One-third of participants said that overprotective mothers were hesitant to leave their children’s side, despite the clinic’s mother-child separation policy for treatment. Participants noticed that some mothers permitted everything that their children demanded. They stated that such mothers did not clean their children’s teeth because they did not like toothbrushing. As a result, they said, children of concern often failed in developing healthy adult teeth.

#### Children with a complicated home environment

All the participants considered that mothers’ home care was essential to maintain oral health among children of concern. However, they felt that they could not expect the children’s mothers with adequate care because such mothers mostly had disabilities, such as autism, intellectual disability, and depression. One-fifth of participants said that there were mothers whose attitudes and communication was considered unconventional. Participants watched out mothers who looked little care for others’ responses—furthermore, participants suspected mothers’ DDs through a conversation.

I do not know for sure. The mother herself has some problems, so-called autism, because I cannot make myself understood when I talk to her (No. 1).

The minority of participants beware of some mothers’ poor dental health literacy in that they believed that caries of deciduous teeth was not a health threat because permanent teeth could replace them. Moreover, a participant (Participant ID No. 17) affirmed a mother who said that her children’s teeth melted since birth. Related to mothers’ low oral health literacy, the minority of participants considered that mothers with many children, complicated marital relationships, social security, and foreign status often could not afford to care for their children’s teeth seriously. They noticed that such mothers also had severe rampant caries. Participants said they had wished to do something for children of concern and their mothers, although they knew they could not do anything except for children’s dental treatment. That is why they awaited that a multidisciplinary team would work to deliver care for the family with heavy burdens.

There were community health nurses and care workers who visited the family once a week and did oral care … because not only the child but also the mother had problems. I was cheered up to hear that story because I knew that not all community healthcare systems were useless (Participant ID No. 11).

## Discussion

The reflections of 21 dentists were studied using grounded theory for accounting for the individual and environmental factors of children of concern. Participants discussed various views, such as children with potential DDs, dental caries possibly derived from neglect, dental phobia possibly derived from trauma, and complicated sociocultural background. This study results provide a firm ground for the early detection of children with possible health risks. Previous studies have shown the ambiguity of the concept of children of concern because this term emphasizes the preventive detection of health risks such as the likelihood of DDs and maltreatment [8–10, 30–32]. However, this study presented more obvious children’s conditions considered health risks-rampant severe caries and dental phobia. Therefore, this study could provide some critical factors that might discern children of concern.

Participants cared for the unusuality of children’s communication and behaviours. They suspected that unusuality were the symptoms of ASD. According to The Diagnostic and Statistical Manual of Mental Disorders (DSM–5), persistent deficits in social communication and social interaction across multiple contexts are factors in the diagnostic criteria of ASD [33]. It is inferred that participants noticed the importance of assessing the quality of social communication and interaction. Although public health nurses watch social communication and interaction conditions at child health checkups, they tend to care for mother-child interaction [9, 34]. However, it is considered difficult for professionals and parents to be aware of children’s social communication and interaction abnormality when a mother and child relationship was satisfactory [8, 9]. Therefore, more than 60% of public health nurses answered that they could not adequately screen children with DDs at 18-month health checkups, according to a nationwide survey [31]. That is why this study’s results contribute to multidisciplinary professionals’ knowledge and practice by vividly describing the social and behavioural characteristics among children of concern.

As for impulsivity and hyperactivity, some participants considered those symptoms stemmed from ASD, and other participants considered ADHD. Previous research revealed that one group of children with diagnosed ASD and the other group of ADHD had similar impulsivity and hyperactivity scores, although ASD group showed significantly higher scores in social communication deficits and repetitive behaviours [35]. Participants’ remarks based on clinical experiences might prove current research trends on ASD and ADHD’s comorbidity. Also, this study results shed a new light on the concept of children of concern in that the possibility of ASD has been primarily paid attention [8]. Future research is considered necessary to thoroughly explore professionals’ perceptions of hyperactivity and impulsivity among children of concern to obtain clues to understand the similarities and differences between ASD and ADHD.

Moreover, this study implied that children of concern are the subject of mental healthcare. Previous studies regarding the perspectives of children of concern among public health nurses and daycare centre teachers have not revealed the possibility of mental health problems other than DDs [8, 9]. A project of providing health checkups at 5-years of age only found that 56 children (5.4%) were diagnosed with DDs and six children were diagnosed with maltreatment during the 8-years study period [32]. However, mental health problems among children and adolescents have a significant influence on their health indicators. The suicide rate under 20 years old is consecutively high in 30 years, and this has been a social concern in Japan [36]. A review suggested that dental phobia including dental anxiety and dental fear was associated with other psychiatric disorders and symptoms and patients with a high level of dental anxiety were more prone to have a high level of comorbid mental health problems [37]. Therefore, this study results illuminated the importance of dental phobia as a marker of mental health problems that were hardly detected in Japan’s currently designed child health checkup systems.

This study could present advanced factors of severe rampant caries and dental neglect for the relationship between children of concern and maltreatment. According to a survey carried out by the Japanese Society for Oral Health, dental caries was significantly more prevalent in children at child consultation centres [38]. Therefore, our findings are in line with past research that suggests a relationship between maltreated children and dental caries. As for neglect, a systematic review indicated it has to be distinguished from poverty circumstances [39]. Our findings also supported the report above. There were children with severe caries due to their mothers’ negligence of children’s oral health needs, even though they lived in a luxury residential area. In a review of dental neglect [39], socioeconomic factors have been little studied. This review pointed out that the difficulties of dealing with poverty as a potential factor of neglect because the cost of child health care is free or reimbursed in Western Europe [39]. In Japan, child dental care costs are also mostly free or reimbursed, depending on the municipalities’ system and the parent’s economic status. Therefore, this study highlights the need further to investigate the influence of socioeconomic factors on dental neglect.

Previous studies indicated that maternal factors are indispensable in detecting children of concern [8, 9, 30]. Our findings are basically in line with previous studies in that mothers’ psychiatric disabilities, and parenting difficulties were pointed out. However, in our analysis, we identified that mothers’ low health literacy was one of the factors that participants define their children of concern. A survey using the European Health Literacy Survey Questionnaire revealed that health literacy in the Japanese population was in general lower than in Europe [40]. Maternal lower health literacy is likely to relate to more flawed service use, management and outcomes of children with chronic conditions [41]. Our findings suggested that mothers of children of concern had poor oral health literacy about children and themselves. Although the national campaign in Japan aimed to increase parents’ knowledge of children’s social communication and interaction [2], little research has targeted the mother’s oral health literacy. Therefore, this study implied that more opportunities should be provided for mothers and professionals to check mothers’ oral health literacy to enhance child health outcomes.

Moreover, dentists in their 20s claimed their anxieties in treating children of concern and informing their mothers of possible developmental difficulties and dental health management necessities. Our findings are in line with previous studies in that public health nurses in their 20s were less confident in their potential of screening for infants with DDs at child health checkups [31]. A previous interview study with public health nurses suggested that providing care for children with ASD and their parents was tailored to parents’ acceptance; therefore, mentoring junior public health nurses and passing experience-based knowledge were necessary [42]. Dentists also have difficulties in treating children with ASD and dentists considered flexibility and multidisciplinary network were the keys to success [20]. Moreover, a review of dental neglect revealed that detecting the risk was challenging and led to the reluctance of reporting cases [39]. Hence, support and research are necessary for professionals to share knowledge and skills and mentor junior professionals to improve the quality of care for children with chronic health risk factors.

Our findings suggested that participants expected multidisciplinary collaboration for professionals and parents to understand and manage the health status of children of concern adequately. As with CSHCN [12], the needs of children of concern are so holistic and diverse that the multidisciplinary team approach was necessary. A project to screen children’s developmental, behaviour problems and improve the school refusal rate included stakeholders: public health nurses; daycare centre teachers; paediatricians; psychologists; school doctors; school teachers and special education teachers [32]. However, previous research indicated that the professionals’ awareness of multidisciplinary collaboration was not always positive [43]. Because public health nurses in Japan tended to pay more attention to child abuse cases, collaboration effort for the support of children with DDs became less in small and middle-sized municipalities [3, 31, 43]. This research indicated that future research was necessary to explore each professional’s perception of collaboration to design more effective collaboration system.

### Limitations of the study

Although this study contributes to the knowledge base on pediatric dental providers who treat children and mothers of concern, it has some limitations. The small study sample limits the transferability or applicability of its outcomes. Qualitative and quantitative research that samples more extensive and more diverse pediatric dentists to determine this study’s external validity is paramount. It is crucial for future research to expand to include other healthcare providers other than dental providers. Furthermore, the study sample is so homogeneous that it is not sufficient to conclude that the results can be applicable to another practice site. The study’s sample was more female-dominant than the gender ratio of the Japanese Society of Pediatric Dentistry members [24]. Comparing the perceptions of children of concern from diverse communities and genders will be indispensable in developing nationwide, culturally responsive practices for children and mothers. Another limitation is researcher bias. The research team worked to address these biases by forming a multidisciplinary team, consulting with a supervisor, and member checking. Moreover, the closeness of the work environment might have produced bias. The second author HF and third author YA were the members of the studied clinic. Although participants were not hesitant to tell their experiences, including their negative feelings toward children of concern, bias could not be excluded when they worried about social desirability. Finally, the impact of the remuneration will be discussed. The amount of offered gift card was justified considering the hourly minimum wage in 2015 was 905 Japanese yen. However, this compensation might encourage the person who was not seriously interested in children of concern to participating. Moreover, this study’s results revealed that the perception of children of concern was not unanimous even among the studied clinic members. Future research is necessary to elucidate and evaluate pediatric dentists’ perception of children with chronic difficulties profoundly and broadly, implementing qualitative and quantitative methods to include specifically selected participants or a broad range of voluntary basis participants.

## Conclusion

This study contributes to knowledge about children with probable ASD, dental phobia, maltreatment, and challenging home environment. Moreover, our findings added to the knowledge about mothers with possible problems in parenting and their health. Our participants’ narratives provide insight and awareness to an understudied population to guide further research and practice in working with clients affected by possible health risk factors. The 21 pediatric dentists represented in this study help the reader better understand how they screen children, with a particular sensitivity to the developmental, emotional, behavioural difficulties and poor dental health.

## Data Availability

The data used and/or analyzed during the current study are available from the corresponding author on reasonable request.

## Abbreviations

DD: Developmental disability
ASD: Autism spectrum disorder
ADHD: Attention deficit hyperactivity disorder
CSHCN: Children with special health care needs
DSM: Diagnostic and Statistical Manual of Mental Disorders
